# Supplementary material for: MetaRibo-Seq measures translation in microbiomes
Source: Nat Commun. 2020 Jun 29;11:3268. doi: 10.1038/s41467-020-17081-z (PMC7324362; doi:10.1038/s41467-020-17081-z)
Supplement: Supplementary file 10 — Supplementary Data 7 [file 41467_2020_17081_MOESM10_ESM.zip › File2/Confidence_High_Taxonomy/290637_out.krona.html]

Javascript must be enabled to view this page.

members
magnitude
magnitudeUnassigned
count
unassigned
taxon
rank

290637\_out

88

88
superkingdom
2

88
phylum
1239

88
class
186801

88
186802
order

186803
family
88

87
841
genus

species
166486

SRS015890\_contig\_number\_19959
1


SRS011084\_contig\_number\_34082SRS011405\_contig\_number\_contig-100\_231.112773SRS013951\_contig\_number\_contig-100\_1240.215065SRS014235\_contig\_number\_34086SRS014412\_contig\_number\_10182SRS014459\_contig\_number\_24113SRS014923\_contig\_number\_44481SRS015065\_contig\_number\_2749SRS015431\_contig\_number\_contig-100\_1874.489692SRS015578\_contig\_number\_16728SRS015663\_contig\_number\_12039SRS015854\_contig\_number\_22711SRS016095\_contig\_number\_contig-100\_16745.96248SRS016335\_contig\_number\_12815SRS016753\_contig\_number\_contig-100\_665.665SRS017433\_contig\_number\_16296SRS018888\_contig\_number\_contig-100\_9223.37042SRS018984\_contig\_number\_27838SRS019068\_contig\_number\_3244SRS019381\_contig\_number\_16750SRS019397\_contig\_number\_4245SRS019445\_contig\_number\_34085SRS019496\_contig\_number\_1162SRS019787\_contig\_number\_contig-100\_826.33194SRS019910\_contig\_number\_6954SRS022137\_contig\_number\_contig-100\_12401.54747SRS023346\_contig\_number\_1088SRS023715\_contig\_number\_contig-100\_2097.2098SRS024388\_contig\_number\_10536SRS042628\_contig\_number\_30253SRS044535\_contig\_number\_contig-100\_24067.68457SRS045713\_contig\_number\_9011SRS046369\_contig\_number\_181SRS046502\_contig\_number\_8600SRS047014\_contig\_number\_contig-100\_9682.189882SRS049896\_contig\_number\_27667SRS049959\_contig\_number\_51606SRS050026\_contig\_number\_2642SRS050752\_contig\_number\_contig-100\_18613.58778SRS050998\_contig\_number\_contig-100\_767.102456SRS056519\_contig\_number\_contig-100\_22920.60114SRS056537\_contig\_number\_contig-100\_8819.171339SRS058770\_contig\_number\_contig-100\_52.157312SRS064276\_contig\_number\_19843SRS075021\_contig\_number\_6240SRS075716\_contig\_number\_contig-100\_14635.14636SRS076804\_contig\_number\_3087SRS077194\_contig\_number\_12339SRS077335\_contig\_number\_3208SRS097889\_contig\_number\_35520SRS097920\_contig\_number\_31015SRS098073\_contig\_number\_11870SRS098827\_contig\_number\_11537SRS103987\_contig\_number\_7259SRS104036\_contig\_number\_contig-100\_4850.44635SRS104311\_contig\_number\_36991SRS104693\_contig\_number\_11769SRS105153\_contig\_number\_21738SRS142890\_contig\_number\_31741SRS143070\_contig\_number\_10636SRS143148\_contig\_number\_26737SRS143417\_contig\_number\_33813SRS143722\_contig\_number\_contig-100\_605.89916SRS143780\_contig\_number\_27188SRS144135\_contig\_number\_18301SRS144183\_contig\_number\_contig-100\_14875.148427SRS144297\_contig\_number\_4223SRS144362\_contig\_number\_24323SRS144506\_contig\_number\_48096SRS144714\_contig\_number\_contig-100\_755.157566SRS145497\_contig\_number\_contig-100\_27962.66141SRS146764\_contig\_number\_contig-100\_1786.180386SRS147271\_contig\_number\_42413SRS147346\_contig\_number\_33839SRS147377\_contig\_number\_contig-100\_795.42248SRS147425\_contig\_number\_1475SRS148091\_contig\_number\_10481SRS148159\_contig\_number\_32030SRS148253\_contig\_number\_contig-100\_40.92894SRS148319\_contig\_number\_2205SRS148784\_contig\_number\_29493SRS149181\_contig\_number\_4180SRS149325\_contig\_number\_contig-100\_8902.8903SRS893259\_contig\_number\_5020SRS893327\_contig\_number\_11674SRS893342\_contig\_number\_8593
species
360807
86

189330
genus
1

species
39486

SRS149784\_contig\_number\_5900
1
